# Intraoperative Imprint Cytology Versus Frozen Section for Sentinel Lymph Node Assessment in Breast Carcinoma: A Prospective Diagnostic Accuracy Study From a Resource-Limited Setting in Eastern India

**DOI:** 10.7759/cureus.112658

**Published:** 2026-07-14

**Authors:** Ambuj Mishra, Manoj K Paswan, Suravi Debnath, Anshu Jamaiyar, Abhirup Shome

**Affiliations:** 1 Pathology, Rajendra Institute of Medical Sciences, Ranchi, IND; 2 Ophthalmology, Narayan Medical College and Hospital, Sasaram, IND

**Keywords:** breast carcinoma, diagnostic accuracy, frozen section, imprint cytology, intraoperative diagnosis, resource-limited settings, sentinel lymph node biopsy, touch imprint

## Abstract

Background

Reliable intraoperative assessment of sentinel lymph nodes (SLN) is critical for surgical decision-making in breast carcinoma. Frozen section (FS) is the standard intraoperative method, but requires cryostat infrastructure that is unavailable in many centers across eastern India. Imprint cytology (IC) is an inexpensive, tissue-preserving alternative; however, locally relevant prospective data to support its use as a standalone intraoperative test have been lacking.

Aim

To compare the diagnostic accuracy of intraoperative IC and FS for SLN evaluation in breast carcinoma, using permanent paraffin histopathology as the reference standard, and to determine whether IC is non-inferior to FS as an intraoperative diagnostic method.

Methodology

A prospective cross-sectional study was conducted over 18 months at a government tertiary care center in Jharkhand, India. A total of 109 consecutive female patients (aged 30-75 years) with histologically confirmed breast carcinoma undergoing sentinel lymph node biopsy (SLNB) were enrolled. Each SLN was evaluated intraoperatively by IC followed by FS; permanent paraffin-section histopathology served as the reference standard. Sensitivity, specificity, positive predictive value (PPV), negative predictive value (NPV), overall accuracy, and the area under the receiver operating characteristic curve (AUC), with 95% confidence intervals (CIs), were calculated. Inter-method agreement was evaluated using McNemar's test and Cohen's kappa coefficient.

Results

Of 109 SLNs, 61 (56.0%) were positive for metastasis on final histopathology. IC yielded a sensitivity of 86.9% (95% CI: 76.2%-93.2%), specificity of 100%, and overall accuracy of 92.7%; FS yielded a sensitivity of 88.5% (95% CI: 78.2%-94.3%), specificity of 100%, and accuracy of 93.6%. The AUC was 0.934 for IC and 0.943 for FS; DeLong paired comparison demonstrated no statistically significant difference (*P* > 0.05). Inter-method concordance was 99.1%, with a Cohen's kappa of 0.982 (*P* < 0.001). All patients had received NACT; false-negative results for both methods involved small residual metastatic deposits showing post-treatment change.

Conclusions

IC demonstrated diagnostic performance statistically non-inferior to FS for intraoperative SLN assessment in breast carcinoma. Given its lower cost, shorter preparation time, complete tissue preservation, and independence from cryostat infrastructure, IC is a valid, evidence-based, and independently deployable intraoperative modality for resource-limited settings.

## Introduction

Breast cancer is the leading malignancy among women worldwide, with an estimated 2.3 million new cases and 666,000 deaths recorded globally in 2022 [1]. In India, breast cancer has overtaken all other female cancers to become the most frequently diagnosed malignancy, accounting for 13.5% of all new cancer cases according to GLOBOCAN 2022 estimates, with a disproportionately high proportion of patients presenting at an advanced stage owing to multifactorial barriers to early detection, including limited access to specialist care in resource-constrained settings [2,3].

Accurate axillary lymph node staging is central to surgical decision-making in breast carcinoma, as nodal status governs prognosis, determines the extent of axillary surgery, and guides adjuvant therapy planning. Sentinel lymph node biopsy (SLNB), introduced into breast cancer management in 1994, has replaced routine axillary lymph node dissection (ALND) as the standard staging procedure in clinically node-negative patients, significantly reducing operative morbidity, including lymphoedema [4]. Intraoperative assessment of the sentinel lymph node (SLN) allows the surgeons to proceed immediately for ALND when nodal metastasis is confirmed.

Frozen section (FS) analysis has historically been the preferred intraoperative method; however, it requires a cryostat machine, specialized technical support, dedicated laboratory infrastructure, along with irreversible tissue loss from the node, and significant cost [5]. Additionally, the ACOSOG Z0011 randomized trial has led to a global de-escalation of routine intraoperative SLN assessment at many high-income centers; however, this de-escalation applies only to carefully selected patients meeting strict eligibility criteria, with one or two positive SLNs undergoing breast-conserving surgery with whole-breast irradiation; criteria that exclude the majority of patients treated at tertiary care hospitals in India, where mastectomy rates are rising, and access to postoperative radiotherapy is limited [6].

Imprint cytology (IC) offers a simpler and more affordable alternative to FS. It requires only standard glass slides, immediate alcohol fixation, rapid hematoxylin and eosin (H&E) staining, and other consumables available at virtually all levels of the healthcare system, while preserving the residual nodal tissue in its entirety for permanent paraffin sections and other required ancillary testing. Importantly, IC eliminates the risk of tissue loss inherent to cryostat processing. A recent systematic review and meta-analysis reported pooled sensitivities of 74% and 78% for IC and FS, respectively, with comparable specificities approaching 100% for both techniques, suggesting that IC can serve as a reliable substitute for FS in resource-limited settings [7]. Individual prospective studies from the Indian subcontinent show similar findings, with IC sensitivities ranging from 83% to 88% with high specificity [8,9].

Despite this, adequate prospective data from India are lacking. The objectives of the present study were as follows: 

Primary objective: To evaluate the diagnostic accuracy of intraoperative IC against permanent paraffin histopathology as the reference standard for SLN assessment in breast carcinoma.

Secondary objective: To compare the diagnostic performance of IC with FS and to establish whether IC is non-inferior to FS as a standalone intraoperative tool in resource-limited settings.

## Materials and methods

Study design and setting

A prospective cross-sectional study was conducted in accordance with the Standards for Reporting of Diagnostic Accuracy (STARD) 2015 guidelines [10]. The study was carried out at the Department of Pathology and the Onco-surgery Unit, Rajendra Institute of Medical Sciences (RIMS), Ranchi. The study period was 18 months, from July 2024 to January 2026.

Ethics statement

The study synopsis received ethical clearance from the Institutional Ethics Committee (IEC), RIMS, Ranchi, as part of the institutional approval process for postgraduate thesis protocols, before commencement of patient enrolment in July 2024. The individual formal approval letter (IEC Registration No. ERC/769/INST/JH/2015/RR-21; Approval Letter No. 84) was subsequently issued on February 01, 2025, as part of the institution's routine documentation process. All procedures were performed in accordance with the ethical standards of the IEC and with the 1964 Declaration of Helsinki and its later amendments. Written informed consent was obtained from all participants in their preferred language before enrolment. Patient confidentiality was maintained throughout by the use of coded identifiers.

Study population

Inclusion Criteria

The study population comprised adult female patients (no eligible male breast carcinoma case presented during the enrolment period) satisfying all of the following: age between 30 and 75 years; histologically confirmed diagnosis of invasive breast carcinoma by core needle biopsy or excision biopsy before enrolment; clinically node-negative axilla (cN0) on preoperative clinical examination and imaging; and scheduled for SLNB as part of planned surgical management following completion of neoadjuvant chemotherapy (NACT). Receipt of NACT before surgery was a requirement for inclusion; all enrolled patients underwent post-NACT intraoperative SLN assessment.

Exclusion Criteria

Patients were excluded if they had a prior diagnosis of biopsy-proven benign breast disease without concurrent malignancy, had previously undergone axillary surgery or radiotherapy to the axilla, were pregnant or lactating (owing to lactational hyperplasia that may mimic metastatic cytomorphology on imprint preparations) at the time of surgery, had a known contraindication to methylene blue dye, or declined to provide written informed consent.

The lower age limit of 30 years was set because invasive breast carcinoma below this age is uncommon and biologically distinct; no eligible patient below 30 years presented during the enrolment period.

All 109 enrolled patients had completed NACT before surgery; no chemotherapy-naïve or upfront-surgery patients were included.

Sample size calculation

The sample size was calculated prospectively using the formula described by Buderer for estimating a diagnostic proportion with a specified precision, where *Zα*/2 = 1.96 (95% confidence level); *P* = 0.87 (anticipated sensitivity of IC based on published literature reporting sensitivities of 83%-88% from the Indian subcontinent); and *L* = 0.07 (acceptable half-width of the 95% confidence interval (CI)) [11]. Substituting these values yielded n ≈ 89, rounded up to 92 to account for potential dropouts. The final enrolment of 109 patients exceeded this minimum, providing adequate statistical power.

Surgical procedure and specimen collection

All surgical procedures were performed under general anesthesia by the same onco-surgical team throughout the study period, ensuring procedural consistency. Sentinel lymph node identification was done by the methylene blue dye-guided mapping technique. Methylene blue dye (4-5 mL of 1% solution) was injected subcutaneously at one to three peritumoral or subareolar sites, followed by gentle massage of the breast tissue for 3-5 minutes to facilitate lymphatic uptake. Lymph nodes that stained blue or were located along a blue-stained lymphatic channel were considered as sentinel nodes and surgically excised.

Each excised SLN was placed immediately in a sterile dry specimen container without fixative and transferred to the Department of Pathology within 15 minutes of excision. The operating surgeon proceeded simultaneously with the definitive breast surgery. Patients whose SLNs were reported positive on intraoperative assessment subsequently underwent completion ALND of levels I and II, with extension to level III where clinically indicated.

Imprint cytology: preparation and staining

On receiving the specimen, the lymph node was bisected longitudinally along its greatest dimension using a sterile scalpel blade. Touch imprints were prepared by making a single, controlled vertical press of each freshly cut nodal surface onto a clean glass slide, purposefully avoiding lateral gliding to preserve cytological clustering. The imprint slide was immediately immersed in 95% ethyl alcohol for 5-6 seconds for rapid fixation. The fixed slide was then processed through a standardized rapid H&E protocol: hematoxylin (2 minutes); 1% acid alcohol (5-6 dips); Scott’s tap water (5-6 dips); eosin (2 minutes); absolute alcohol (5-6 dips); xylene (2 minutes); coverslipping with DPX mountant. The entire IC preparation and staining process was completed within 10-12 minutes of specimen receipt.

Stained IC slides were examined under light microscopy and independently reported by two senior pathologists, each with more than ten years of experience in surgical pathology and cytopathology, in a blinded sequential manner.

Frozen section: preparation and staining

Following IC slide preparation, the remaining bisected lymph node tissue was transferred immediately to the cryostat room. The Leica cryostat was pre-set to a chamber temperature of −18 to −24 °C. The tissue was mounted on a metallic chuck using optimal cutting temperature (OCT) compound and allowed to freeze for 8-10 minutes. Multiple serial transverse sections were cut at 5-7 µm thickness and transferred onto glass slides. Multiple levels were taken from each node to minimize sampling error. Frozen section slides were stained by the same rapid H&E protocol as described for IC, with the entire process completed within 20-25 minutes of specimen receipt. FS slides were then independently reported by the same two senior pathologists after completion of the IC report, maintaining strict blinding to the IC result.

Histopathological processing

Following intraoperative assessments, all residual nodal tissue was fixed in 10% neutral buffered formalin for a minimum of six hours. Fixed tissue was processed through a standard automated tissue processor, embedded in paraffin wax, and serial sections were cut at 3-5 µm on a rotary microtome and stained with routine H&E; multiple levels were examined from each node. In cases of discordance between intraoperative and initial permanent section results, deeper levels were cut. Permanent paraffin-section histopathology, reported by the consultant pathologist, served as the definitive reference standard. Histological subtyping and grading were performed according to the WHO Classification of Tumors of the Breast, 5th Edition (2022), using the modified Scarff-Bloom-Richardson grading system [12].

Reporting and blinding

A strict sequential blinding protocol was maintained throughout the study to prevent observer bias. The two senior pathologists independently examined and reported IC slides first, without knowledge of each other’s diagnoses or of any clinical details beyond the basic request form. They then independently examined and reported FS slides, maintaining blinding to IC results. The consultant pathologist subsequently reported the permanent paraffin sections without access to any intraoperative diagnoses. In case of discordant opinions between the two reporting pathologists, the final diagnosis was determined by the consultant pathologist on slide review.

Statistical analysis

All data were entered and managed in Microsoft Excel 2019 and analyzed using Jamovi (version 2.6.44; The Jamovi Project, 2024). Continuous variables are reported as mean ± standard deviation (SD) or median with interquartile range (IQR). Categorical variables are reported as frequencies and percentages. The Shapiro-Wilk test was used to assess normality. Between-group comparisons of continuous variables were performed using the independent t-test or Mann-Whitney U test, as appropriate; comparisons across more than two groups used the Kruskal-Wallis H test followed by Dwass-Steel-Critchlow-Fligner post hoc pairwise comparisons.

The diagnostic performance of IC and FS was evaluated against permanent histopathology as the reference standard. Sensitivity, specificity, positive predictive value (PPV), negative predictive value (NPV), overall diagnostic accuracy, positive likelihood ratio (LR+), and negative likelihood ratio (LR-) were calculated with 95% CIs by the Wilson score method. Receiver operating characteristic (ROC) curve analysis was performed, and the area under the curve (AUC) was calculated for each method with 95% CIs; AUCs were compared using the DeLong paired method. IC was considered non-inferior to FS when no statistically significant AUC difference was observed (DeLong *P* > 0.05) with substantial overlap of 95% CIs. Inter-method agreement was assessed using McNemar's test and Cohen's kappa (κ), interpreted on the Landis and Koch scale. A two-tailed *P*-value < 0.05 was considered statistically significant.

## Results

Clinicopathological profile

A total of 109 female patients with histologically confirmed breast carcinoma undergoing intraoperative SLNB were enrolled. Patient age ranged from 30 to 75 years, with a mean of 52.2 ± 11.6 years. The Shapiro-Wilk test (*P* = 0.021) indicated a slight departure from normality in the age distribution. Most patients were in the 51-60 years age group (31, 28.4%), followed by the 41-50 years age group (27, 24.8%) (Table 1). The primary tumor showed a nearly equal laterality distribution: the left breast in 55 (50.5%) and the right breast in 54 (49.5%) of cases. A family history of breast malignancy was documented in only 2 (1.8%) patients (Table 1).

**Table 1:** Clinicopathological profile of the study population (N = 109). IC-NST, invasive carcinoma of no special type; ILC, invasive lobular carcinoma

Characteristic	Category	*n* (%)
Age group (years)	30-40	22 (20.2%)
	41-50	27 (24.8%)
	51-60	31 (28.4%)
	61-75	29 (26.6%)
Tumor laterality	Left	55 (50.5%)
	Right	54 (49.5%)
Histological subtype	IC-NST	101 (92.7%)
	ILC	8 (7.3%)
Tumor grade (IC-NST)	Grade 1	3 (3.0%)
	Grade 2	49 (48.5%)
	Grade 3	49 (48.5%)
Tumor grade (ILC)	Grade 1	1 (12.5%)
	Grade 2	6 (75.0%)
	Grade 3	1 (12.5%)
Family history of breast cancer	Present	2 (1.8%)
	Absent	107 (98.2%)

Invasive carcinoma of no special type (IC-NST) was the predominant histological subtype, accounting for 101 (92.7%) of cases; invasive lobular carcinoma (ILC) constituted the remaining 8 (7.3%). IC-NST cases were predominantly of intermediate Grade 2 (49, 48.5%) or high Grade 3 (49, 48.5%), with only three well-differentiated Grade 1 tumors. ILC cases were predominantly Grade 2 (6, 75.0%), with one each of Grade 1 and Grade 3 (Table 1). Overall, Grade 2 and Grade 3 tumors together comprised 96.3% of the study population.

Age and tumor grade

A Kruskal-Wallis H test demonstrated a statistically significant association between patient age and histological grade (*H* = 7.93, *P* = 0.019; η² = 0.073). Post hoc pairwise comparisons by the Dwass-Steel-Critchlow-Fligner (DSCF) test revealed that patients with Grade 1 tumors (mean age 37.2 years) were significantly younger than those with Grade 2 (mean age 51.5 years; *P* = 0.047) and Grade 3 tumors (mean age 54.3 years; *P* = 0.015). No significant age difference was found between the Grade 2 and Grade 3 groups (*P* = 0.558). The association between age, tumor grade, and SLN positivity is summarized in Table 2.

**Table 2:** Association of patient age and tumor grade with SLN positivity. Statistical test: Pearson's chi-square (χ²) test for the association between each variable and SLN positivity. SLN, sentinel lymph node

Variable	Category	SLN positive, *n* (%)	SLN negative, *n* (%)	Total	Test statistic	*P*-value
Age group (years)	30-40	11 (50.0%)	11 (50.0%)	22	χ² = 7.34, df = 3	0.062
	41-50	19 (70.4%)	8 (29.6%)	27		
	51-60	20 (64.5%)	11 (35.5%)	31		
	61-75	11 (37.9%)	18 (62.1%)	29		
	Subtotal	61 (56.0%)	48 (44.0%)	109		
Tumor grade	Grade 1	2 (50.0%)	2 (50.0%)	4	χ² = 44.34, df = 2	<0.001
	Grade 2	14 (25.5%)	41 (74.5%)	55		
	Grade 3	45 (90.0%)	5 (10.0%)	50		
	Subtotal	61 (56.0%)	48 (44.0%)	109		

SLN positivity

On final histopathological examination, 61 of 109 SLNs (56.0%) demonstrated metastatic involvement, while 48 (44.0%) were negative. Age did not differ significantly between the SLN-positive group (mean 51.3 ± 11.1 years) and the SLN-negative group (mean 53.4 ± 12.4 years); both the independent sample t-test (t = −0.937, P = 0.351) and the Mann-Whitney U test (U = 1285.5, P = 0.277) showed no significant association.

Intraoperative assessment by IC

IC identified metastatic deposits in 53 of 109 nodes (48.6%), with 56 (51.4%) reported as negative. Against final histopathology, IC yielded 53 true positives, 48 true negatives, 0 false positives, and 8 false negatives (χ² = 77.74, df = 1, *P* < 0.001; Table 3). No false-positive results were recorded. All eight false-negative cases involved IC-NST (five Grade 3 and three Grade 2); the likely explanation is post-treatment hypocellularity of the metastatic deposits with stromal fibrosis, resulting in under-sampling of residual tumor cells on the imprint slide. The sensitivity of IC was 86.9% (95% CI: 76.2%-93.2%), specificity 100.0% (95% CI: 92.6%-100.0%), PPV 100.0% (95% CI: 93.2%-100.0%), NPV 85.7% (95% CI: 74.3%-92.6%), and overall diagnostic accuracy 92.7% (95% CI: 86.2%-96.2%) (Table 4).

**Table 3:** Contingency tables for IC and FS against permanent histopathology. Statistical analysis: Chi-square (χ²) test. Panel A (IC): χ² = 77.74, df = 1, *P* < 0.001. Panel B (FS): χ² = 80.71, df = 1, *P* < 0.001. TP, true positive; FP, false positive; FN, false negative; TN, true negative; IC, imprint cytology; FS, frozen section

Parameters	Histology positive	Histology negative	Total	Test statistic	*P*-value
Panel A: Imprint cytology					
IC positive	53 (TP)	0 (FP)	53	χ² = 77.74, df = 1	<0.001
IC negative	8 (FN)	48 (TN)	56		
Total	61	48	109		
Panel B: Frozen section					
FS positive	54 (TP)	0 (FP)	54	χ² = 80.71, df = 1	<0.001
FS negative	7 (FN)	48 (TN)	55		
Total	61	48	109		

**Table 4:** Diagnostic performance metrics for imprint cytology and frozen section. 95% CIs calculated by the Wilson score method; AUC comparison by the DeLong paired method. PPV, positive predictive value; NPV, negative predictive value; LR+, positive likelihood ratio; LR-, negative likelihood ratio; AUC, area under the ROC curve; CI, confidence interval

Parameter	Imprint cytology (95% CI)	Frozen section (95% CI)
Sensitivity	86.9% (76.2%-93.2%)	88.5% (78.2%-94.3%)
Specificity	100.0% (92.6%-100.0%)	100.0% (92.6%-100.0%)
PPV	100.0% (93.2%-100.0%)	100.0% (93.4%-100.0%)
NPV	85.7% (74.3%-92.6%)	87.3% (76.0%-93.7%)
Overall Accuracy	92.7% (86.2%-96.2%)	93.6% (87.3%-96.9%)
LR+	Undefined (100% specificity)	Undefined (100% specificity)
LR−	0.131	0.115
AUC (ROC)	0.934 (0.892-0.977)	0.943 (0.902-0.983)
Youden Index	0.869	0.885

Intraoperative assessment by FS

FS reported 54 nodes as positive (49.5%) and 55 as negative (50.5%). Against final histopathology, FS yielded 54 true positives, 48 true negatives, 0 false positives, and 7 false negatives (Table 3; χ² = 80.71, df = 1, P < 0.001). All seven false-negative FS cases were IC-NST (five Grade 3 and two Grade 2). No false-positive results were observed. The sensitivity of FS was 88.5% (95% CI: 78.2%-94.3%), specificity 100.0% (95% CI: 92.6%-100.0%), PPV 100.0% (95% CI: 93.4%-100.0%), NPV 87.3% (95% CI: 76.0%-93.7%), and diagnostic accuracy 93.6% (95% CI: 87.3%-96.9%) (Table 4). Since both methods achieved 100% specificity, the positive likelihood ratio (LR+) was mathematically undefined for both, indicating that a positive intraoperative result constituted a perfect predictor of true nodal metastasis. The negative likelihood ratio (LR-) was 0.131 for IC and 0.115 for FS.

Grade-stratified diagnostic performance

The diagnostic performance of both methods across histological grades is summarized in Table 5. In Grade 1 tumors (*n* = 4), both IC and FS achieved 100% sensitivity, specificity, and accuracy. In Grade 2 tumors (*n* = 55), IC sensitivity was 78.6%, and FS sensitivity was 85.7%, with both maintaining 100% specificity and overall accuracies of 94.5% and 96.4%, respectively. In Grade 3 tumors (*n* = 50), of which 45 were SLN-positive, both IC and FS demonstrated identical performance: sensitivity 88.9%, specificity 100.0%, and overall accuracy 90.0%. The marginally lower sensitivity observed in Grade 2 tumors relative to Grade 3 tumors is attributable to the smaller size of metastatic deposits and the more pronounced treatment effect of NACT in that subgroup.

**Table 5:** Grade-stratified diagnostic performance of IC and FS. Grade refers to the modified Scarff-Bloom-Richardson (Elston-Ellis) grading system. SLN+, sentinel lymph node positive for metastasis; IC, imprint cytology; FS, frozen section

Grade	n	SLN+	IC sensitivity	IC accuracy	FS sensitivity	FS accuracy	Specificity (Both)	χ² (IC)	χ² (FS)	*P*-value
Grade 1	4	2	100%	100%	100%	100%	100%	1	1	0.317
Grade 2	55	14	78.60%	94.50%	85.70%	96.40%	100%	35.51	40.07	<0.001
Grade 3	50	45	88.90%	90.00%	88.90%	90.00%	100%	17.01	17.01	<0.001

Comparative performance: IC versus FS

Direct comparison of the two intraoperative methods demonstrated near-identical diagnostic performance. Of 109 cases, 108 (99.1%) received concordant diagnoses: 53 positive and 55 negative by both techniques. A single discordant case (0.9%) was identified: a 41-year-old patient with Grade 2 IC-NST who had received NACT, in whom FS correctly identified nodal metastasis while IC was falsely negative; final histopathology confirmed SLN positivity. McNemar's test on this single discordant pair yielded χ² = 0.00 (*P* = 1.000), confirming no statistically significant difference in diagnostic categorization between the two methods. Cohen's kappa coefficient was 0.982 (*z* = 10.3, *P* < 0.001), representing almost perfect inter-method agreement (Table 4).

ROC analysis

**Figure 1:**
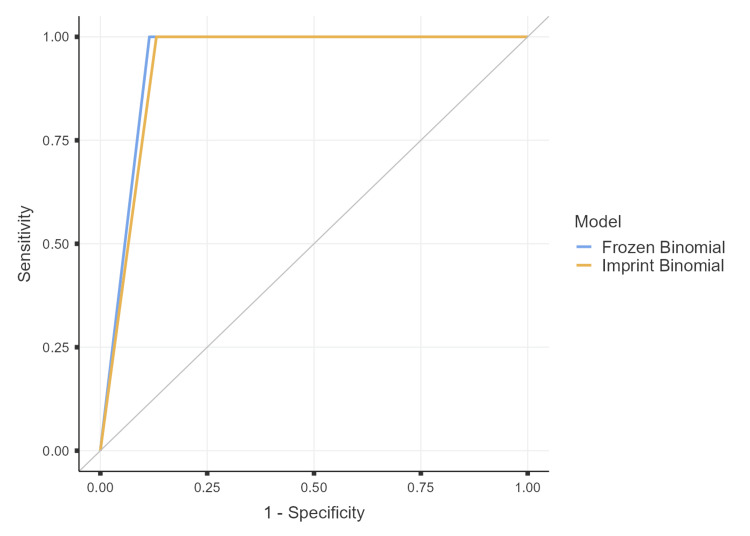
Receiver operating characteristic (ROC) curves for imprint cytology and frozen section against permanent histopathology as the reference standard. Area under the curve (AUC): imprint cytology = 0.934 (95% CI: 0.892-0.977); frozen section = 0.943 (95% CI: 0.902-0.983).

ROC curve analysis confirmed excellent discriminatory performance for both intraoperative techniques (Table 4). IC demonstrated an AUC of 0.934 (95% CI: 0.892-0.977; *P* < 0.001), while FS showed an AUC of 0.943 (95% CI: 0.902-0.983; *P* < 0.001), as shown in Figure 1. The Youden index was 0.869 for IC and 0.885 for FS, with both methods operating close to the ideal classification point on the ROC curve. Comparison of the two AUCs by the DeLong paired method demonstrated no statistically significant difference (*P* > 0.05). The wide overlap of the 95% CIs formally establishes the non-inferiority of IC relative to FS for intraoperative SLN assessment in breast carcinoma.

## Discussion

SLNB is the standard approach for axillary staging in clinically node-negative breast carcinoma, and the accuracy of intraoperative SLN assessment determines the need for immediate completion of ALND. The present prospective study demonstrates that IC achieves diagnostic performance equivalent to FS, with near-perfect concordance (*κ* = 0.982), identical 100% specificity, and comparable sensitivity (IC: 86.9% vs. FS: 88.5%) and overall accuracy (IC: 92.7% vs. FS: 93.6%). These findings are derived from a real-world government tertiary care setting in eastern India, filling an important geographic and institutional gap in the existing literature.

The SLN positivity rate of 56.0% in our cohort is higher than that reported in many Western series, reflecting the predominance of Grade 2 and Grade 3 tumors (96.3% of cases) and advanced stage at presentation. Despite universal neoadjuvant chemotherapy, residual nodal metastasis remained common, consistent with the low pathological complete-response rates and the higher-grade, late-presenting tumors characteristic of Indian and Asian cohorts.

The sensitivity of 86.9% for IC in our study is superior to the pooled sensitivity of 74% reported for IC in the most recent systematic review and meta-analysis by Bharath et al., and compares favorably with the 83%-88% sensitivities reported from the Indian subcontinent by Khanna et al. and Hashmi et al. [7-9]. Our FS sensitivity of 88.5% aligns closely with the pooled FS sensitivity of 83%-90% reported in large meta-analyses. The superior performance of both methods relative to several published series is attributable to the standardized protocol, including multiple-level sectioning for FSs, which minimizes sampling error from eccentric or small-volume metastatic deposits. The 100% specificity achieved by both methods is consistent with the literature and is of critical practical importance: it ensures that no patient in our cohort was subjected to an unnecessary completion ALND based on a false-positive intraoperative result.

The false-negative results - eight for IC and seven for FS - all involved small residual metastatic deposits in nodes exhibiting chemotherapy-induced treatment effect. As the entire cohort was neoadjuvant-treated, these reflect the recognized difficulty of detecting sparse post-treatment residual tumor cells rather than any difference between treated and untreated patients. This finding is clinically significant and mechanistically consistent. Neoadjuvant chemotherapy induces treatment effect within metastatic deposits, characterized by tumor cell apoptosis, stromal fibrosis, mucin accumulation, and replacement of viable tumor cells by histiocytes and fibroblasts, resulting in post-treatment hypocellularity and architectural distortion that reduces the yield of both IC and FS. Similar findings have been reported by Grabenstetter et al., who identified neoadjuvant chemotherapy-related histomorphology changes as a principal factor underlying false-negative intraoperative FS results for SLNs [13].

ILC represents a recognized diagnostic challenge for both IC and FS due to its discohesive single-file infiltration pattern and bland cytomorphology. Although our ILC subgroup was small (*n* = 8) and precludes formal subgroup analysis, the literature consistently reports lower IC sensitivities for ILC, with values as low as 33%-40% in dedicated series. Clinicians and pathologists should be aware of this limitation when interpreting intraoperative results in patients with ILC.

The ACOSOG Z0011 trial has established that selected patients, those with one or two positive SLNs, undergoing breast conserving surgery with whole-breast radiotherapy, do not benefit from completion ALND [6]. However, the majority of patients at government tertiary care hospitals in India do not fulfil these criteria: mastectomy rates are high, access to postoperative radiotherapy is variable, and re-admission for a second surgical procedure imposes a disproportionate financial and logistical burden on patients from rural and peri-urban areas. In this clinical context, intraoperative SLN assessment retains its full clinical utility, and the ability to perform it without a cryostat using IC alone has direct practical implications for the approximately 600 government medical colleges and district hospitals across India, where cryostat availability cannot be guaranteed.

IC carries significant practical advantages over FS in resource-limited settings. The entire IC preparation and staining process is completed within 10-12 minutes of specimen receipt, compared to 20-25 minutes for FS, offering a meaningful reduction in operative time. The consumables for IC - glass slides, alcohol, H&E stains, and DPX mountant - are substantially less expensive than those required for FS (OCT compound, cryostat blade replacements, and machine maintenance). Crucially, IC leaves the entire lymph node intact for permanent section processing, whereas FS invariably results in irreversible tissue loss that may compromise final histological assessment. For high-volume public hospitals, IC is not only cost-effective but also an evidence-based choice that preserves tissue integrity [14].

The non-inferiority of IC relative to FS in the present study is formally established by the DeLong paired AUC comparison (*P* > 0.05) and the near-identical and widely overlapping 95% CIs of the two AUCs (IC: 0.892-0.977; FS: 0.902-0.983). The almost perfect Cohen’s kappa of 0.982 exceeds the range of 0.85-0.92 reported in comparable studies [1] and confirms that substituting IC for FS in routine practice would result in reclassification of fewer than 1% of cases.

Limitations

The present study has several limitations. First, this was a single-center study, which may limit generalizability; a multicenter prospective validation study would strengthen external validity. Second, sentinel node identification was performed exclusively using methylene blue dye-guided mapping without radioisotope-labelled colloid or fluorescence-based techniques; the SLN identification rate and false-negative rate of the surgical technique itself were not formally tracked. Third, turnaround time for IC and FS was not recorded prospectively for each case, preventing a precise quantitative comparison of intraoperative reporting speed. Fourth, sub-classification of metastatic deposits into isolated tumor cells, micrometastases, and macrometastases per AJCC/UICC staging criteria was not performed for all cases, limiting method-specific performance analysis by deposit size. Fifth, the ILC subgroup (*n* = 8) was too small for meaningful subgroup-specific performance analysis. Sixth, formal inter-observer agreement between the two reporting pathologists was not quantified separately for each method. Finally, no long-term oncological follow-up data are available; the clinical impact of false-negative intraoperative results on axillary recurrence rates could not be assessed.

Representative photomicrographs of the intraoperative preparations could not be provided, as slides were not archived for retrospective imaging; illustrative images would strengthen future reports.

As the entire cohort was neoadjuvant-treated, the reported sensitivities apply specifically to the post-NACT setting; treatment-induced nodal fibrosis and hypocellularity may lower intraoperative yield, and the baseline sensitivity of IC and FS in chemotherapy-naïve patients cannot be inferred from these data and warrants separate evaluation.

## Conclusions

Both IC and FS are highly accurate, specific, and reliable methods for intraoperative SLN assessment in breast carcinoma. IC achieves diagnostic performance statistically non-inferior to FS, with 100% specificity, near-perfect concordance, and comparable sensitivity and AUC. Given its significantly lower cost, shorter preparation time, complete tissue preservation, and independence from cryostat infrastructure, IC represents a valid, evidence-based, and independently deployable intraoperative modality. For high-volume government medical colleges and district hospitals in India and similarly resource-limited settings worldwide, routine adoption of IC as the primary or standalone intraoperative tool for SLN assessment in breast carcinoma is scientifically justified and clinically recommended.
